# Supplementary material for: The impact of poly-A microsatellite heterologies in meiotic recombination
Source: Life Sci Alliance. 2019 Apr 25;2(2):e201900364. doi: 10.26508/lsa.201900364 (PMC6485458; doi:10.26508/lsa.201900364)
Supplement: Supplementary file 1 [file LSA-2019-00364_Supplement_Data1.docx]

**Supplement**

**Who is in the driver’s seat? The impact of poly-A microsatellite heterology in meiotic recombination**

Angelika Heissl^1^, Andrea J. Betancourt^2^, Philipp Hermann^3^, Gundula Povysil^4,*^, Barbara Arbeithuber^1,&^, Andreas Futschik^3^, Thomas Ebner^5^, Irene Tiemann-Boege^1^

**Supplement content:**

*Supplement material and methods:*

[1. PCR conditions for flanking SNP genotyping 34](#_Toc525826554)

[2. PCR conditions for haplotyping 35](#_Toc525826555)

[3. PCR conditions, purification and sequencing primers for PRDM9 variant identification 36](#_Toc525826556)

[4. Pooled sperm typing 37](#_Toc525826557)

[5. PCR conditions for CO internal SNP genotyping with TaqMan 39](#_Toc525826558)

[6. PCR conditions for CO and NCO internal SNP genotyping with allele-specific primers 40](#_Toc525826559)

[7. Testing the number of amplifiable genomes 43](#_Toc525826560)

[8. Data analysis 44](#_Toc525826561)

*Supplement tables:*

[Supplement Table S1. Donor genotypes and haplotypes 47](#_Toc525826564)

[Supplement Table S2. Crossover and non-crossover frequency estimation. 48](#_Toc525826565)

[Supplement Table S3. Crossover and non-crossover centers. 49](#_Toc525826566)

[Supplement Table S4. CO per reciprocal 50](#_Toc525826567)

[Supplement Table S5. NCOs per reciprocal 51](#_Toc525826568)

[Supplement Table S6. NCOs: Simple- , co- and complex conversions 52](#_Toc525826569)

[Supplement Table S7. Conversion tract length in non-crossover events 53](#_Toc525826570)

[Supplement Table S8. Frequency of complex events per amplifiable sperm 54](#_Toc525826571)

[Supplement Table S9. Complex Crossovers 55](#_Toc525826572)

[Supplement Table S10. Poly-A mutation frequencies 57](#_Toc525826573)

[Supplement Table S11. Correction factors 58](#_Toc525826574)

SI Material and Methods

# ****PCR conditions for flanking SNP genotyping****

**We first identified a set of informative donors, which were heterozygous for all four SNPs flanking the HSII region:** rs7201177 C/G, rs1861187 C/T, rs4786854 C/T and rs4786855 A/C**. For simplicity only the last three digits of the SNP ID plus the allele types are used throughout this work. Hundreds of different DNA samples extracted from human sperm were genotyped for the four chosen SNPs. For this purpose, allele-specific primers for each SNP and a universal primer (open forward OF or open reverse OR) were designed as described in full detail previously (**[**Heissl et al., 2017**](#_ENREF_37)**,** [**Arbeithuber et al., 2015**](#_ENREF_4)**). Primer sequences are listed in the table below, with** allele-specific bases shown in green and mismatches in red. The last three 3´ bases of the allele-specific primers are synthesized with phosphorothioate (PTO) bonds (represented in the primer sequence as asterisks) to increase the specificity of the primers. Numbers in brackets after the primer sequence represent the primer length. Primers were ordered lyophilized at Eurofins genomics and solved in molecular biology grade water (Sigma Aldrich).


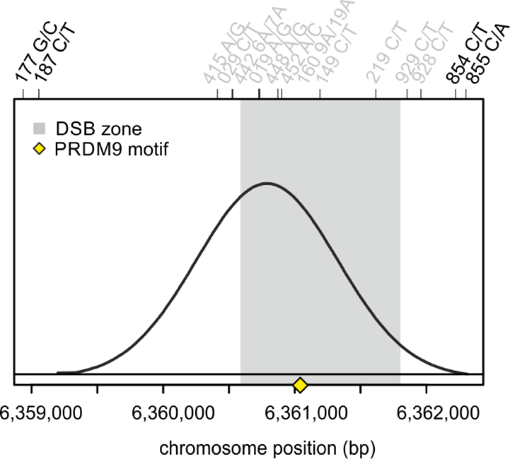


***PCR primers:***

| **SNP ID** | **SNP position**  **(GRCh37/hg19)** | **Primer ID** | **Sequence 5´- 3´** | **Amplicon length [bp]** | **Annealing temp. [°C]** | **Polymerase** |
| --- | --- | --- | --- | --- | --- | --- |
| rs7201177  C/G | 6,358,968 | F-7201177(C) | TAG GAC GTC TCT CTG C*T*T*C (19) | 88 | 63 | OneTaq (NEB) |
|  |  | F-7201177(G) | TAG GAC GTC TCT CTG C*T*T*G (19) |  |  |  |
|  |  | OR-7201177 | CTG GGT ATA GGG TGA GAG GA (20) |  |  |  |
| rs1861187  C/T | 6,359,077 | F-1861187(C) | GCG ATT GAA ATA ATC AGG TC*T*C*A*C (24) | 69 | 63 | OneTaq (NEB) |
|  |  | F-1861187(T) | GCG ATT GAA ATA ATC AGG TC*T*C*A*T (24) |  |  |  |
|  |  | OR-1861187 | GAA TTC AAA ACA GGC GAA CG (20) |  |  |  |
| rs4786854  C/T | 6,362,280 | OF-4786854 | CCA ACT TAG GTG AGG CAC CA (20) | 215 | 63 | OneTaq (NEB) |
|  |  | R-4786854(C) | CCT TGT TTC CTG ATT ACA C A*G*C*G (23) |  |  |  |
|  |  | R-4786854(T) | CCT TGT TTC CTG ATT ACA C A*G*C*A (23) |  |  |  |
| rs4786855  A/C | 6,362,356 | OF-4786855 | CCA GGA AGA ACC AGC ATT TC (20) | 127 | 63 | OneTaq (NEB) |
|  |  | R-4786855(A) | GAA GTA GCA ATG AGA GAG AGA AG*A*A*T (26) |  |  |  |
|  |  | R-4786855(C) | GAA GTA GCA ATG AGA GAG AGA AG*A*A*G (26) |  |  |  |

| **Step** | **Temp.** | **Time** |  |
| --- | --- | --- | --- |
| **1** | **95°C** | 2 min |  |
| **2** | **95°C** | 15 sec | Go to step 2 for 45x |
| **3** | **63°C** | 10 sec |  |
| **4** | **68°C** | 15 sec (plate read) |  |
| **5** | **78°C** | 5 sec (plate read) |  |
| **6** | **72°C** | 7 min |  |
| **7** | **65-95°C** | 5 sec / 0.5°C | Melt curve analysis |

# ****PCR conditions for haplotyping****

**To perform pooled sperm typing assays, the haplotypes of each donor were determined as described in (**[**Arbeithuber et al., 2015**](#_ENREF_4)**,** [**Arbeithuber et al., 2017**](#_ENREF_5)**). In short, the allele-specific primers of SM-Material and Methods 1, also used for the genotyping assays, were mixed in four different combinations (in-in, out-out, out-in and in-out) in a total of 16 reactions. Note that the phase of the alleles could be inferred already with 12 reactions. Here we used only allele-specific primers.**

***PCR primer combinations:***

| **combination** | **SNP ID** | **Primer sequence forward 5´- 3´** | **Primer sequence reverse 5´- 3´** | **Amplicon length [bp]** |
| --- | --- | --- | --- | --- |
| in-in | 187C + 854C | GCG ATT GAA ATA ATC AGG TC*T*C*A*C | CCT TGT TTC CTG ATT ACA C A*G*C*G | 3205 |
|  | 187C + 854T | GCG ATT GAA ATA ATC AGG TC*T*C*A*C | CCT TGT TTC CTG ATT ACA C A*G*C*A |  |
|  | 187T + 854C | GCG ATT GAA ATA ATC AGG TC*T*C*A*T | CCT TGT TTC CTG ATT ACA C A*G*C*G |  |
|  | 187T + 854T | GCG ATT GAA ATA ATC AGG TC*T*C*A*T | CCT TGT TTC CTG ATT ACA C A*G*C*A |  |
| out-out | 177C + 855C | TAG GAC GTC TCT CTG C*T*T*C | GAA GTA GCA ATG AGA GAG AGA AG*A*A*G | 3390 |
|  | 177C + 855A | TAG GAC GTC TCT CTG C*T*T*C | GAA GTA GCA ATG AGA GAG AGA AG*A*A*T |  |
|  | 177G + 855C | TAG GAC GTC TCT CTG C*T*T*G | GAA GTA GCA ATG AGA GAG AGA AG*A*A*G |  |
|  | 177G + 855A | TAG GAC GTC TCT CTG C*T*T*G | GAA GTA GCA ATG AGA GAG AGA AG*A*A*T |  |
| in-out | 187C + 855C | GCG ATT GAA ATA ATC AGG TC*T*C*A*C | GAA GTA GCA ATG AGA GAG AGA AG*A*A*G | 3281 |
|  | 187C + 855A | GCG ATT GAA ATA ATC AGG TC*T*C*A*C | GAA GTA GCA ATG AGA GAG AGA AG*A*A*T |  |
|  | 187T + 855C | GCG ATT GAA ATA ATC AGG TC*T*C*A*T | GAA GTA GCA ATG AGA GAG AGA AG*A*A*G |  |
|  | 187T + 855A | GCG ATT GAA ATA ATC AGG TC*T*C*A*T | GAA GTA GCA ATG AGA GAG AGA AG*A*A*T |  |
| out-in | 177C + 854C | TAG GAC GTC TCT CTG C*T*T*C | GAA GTA GCA ATG AGA GAG AGA AG*A*A*G | 3314 |
|  | 177C + 854T | TAG GAC GTC TCT CTG C*T*T*C | GAA GTA GCA ATG AGA GAG AGA AG*A*A*T |  |
|  | 177G + 854C | TAG GAC GTC TCT CTG C*T*T*G | GAA GTA GCA ATG AGA GAG AGA AG*A*A*G |  |
|  | 177G + 854T | TAG GAC GTC TCT CTG C*T*T*G | GAA GTA GCA ATG AGA GAG AGA AG*A*A*T |  |

***PCR conditions:***

| **Step** | **Temp.** | **Time** |  |
| --- | --- | --- | --- |
| **1** | **92°C** | 2 min |  |
| **2** | **92°C** | 10 sec | Go to step 2 for 45x |
| **3** | **57°C** | 15 sec |  |
| **4** | **68°C** | 4 min 30sec (plate read) |  |
| **5** | **80°C** | 5 sec (plate read) |  |
| **6** | **72°C** | 5 min |  |
| **7** | **65-95°C** | 5 sec / 0.5°C | Melt curve analysis |

# ****PCR conditions, purification and sequencing primers for PRDM9 variant** **identification****

**All analyzed donors were of European descent,** **and likely carriers of PRDM9-A or B (homozygous or heterozygous). Both variants differ in position 898 of Exon 10, where PRDM9-A carries a G and PRDM9-B carries a C (**[**Patel et al., 2017**](#_ENREF_77)**,** [**Berg et al., 2010**](#_ENREF_12)**,** [**Pratto et al., 2014**](#_ENREF_84)**).**

For the identification of the PRDM9 variants in each donor, Prdm9 exon 10 was amplified. A total reaction volume of 50 µl containing **1 U Phusion HSII (Thermo Scientific),** 1x HF Buffer (Biozym), 0.2 mM dNTPs (Biozym), 0.5 µM forward primer, 0.5 µM reverse primer (both Eurofins), and 20 ng genomic DNA were used for each PCR reaction. PCR products were purified with PEG and sequenced with standard capillary sequencing. The PCR products were cleaned up via PEG purification and sequenced at LGC genomics, Germany.

***PCR primers:***

Numbers in brackets after the primer sequence represent the primer length.

| **Primer ID** | **Sequence 5´- 3´** | **Amplicon length [bp]** | **Annealing temp. [°C]** | **Polymerase** |
| --- | --- | --- | --- | --- |
| hP9-Ex10_F1 | TGA GGT TAC CTA GTC TGG CA (20) | 1959 | 61 | Phusion U (Thermo Fisher Scientific) |
| hP9-3UTR_R2 | ATG AAA GTG GCG GAT TTG TTT A (22) |  |  |  |

***PCR conditions:***

| **Step** | **Temp.** | **Time** |  |
| --- | --- | --- | --- |
| **1** | **94°C** | 30 sec |  |
| **2** | **94°C** | 5 sec | Go to step 2 for 35x |
| **3** | **61°C** | 5 sec |  |
| **4** | **72°C** | 30 sec |  |
| **5** | **72°C** | 5 min |  |
| **6** | **12°C** | hold |  |

***PCR clean up with PEG***

**For each 50** **μl PCR reaction, 2.5** **μl Co-Precipitant Pink (BIOLINE) was added and mixed with 2.5 volumes Fast-PEG solution (**10% Polyethylenglycol 8000 (Carl ROTH), 1 M NaCl and 30% Isopropanol). After 5 min incubation, the sample was centrifuged with 16,000 x g for 5 min and the supernatant discarded. The pellet was washed with 200 **μl 75% ethanol, followed by centrifugation at 16,000 x g for 5 min. The supernatant was discarded once more and the pellet was dried for 5 min. The DNA pellet was dissolved in 25 μl** 5 mM Tris-HCl, pH 8.5.

***Sequencing primer:***

| **Primer ID** | **Sequence 5´- 3´** |
| --- | --- |
| hP9ZnF_seqLGC_R | GGG AAT ATA AGG GGT CAG CAG (21) |

# ****Pooled sperm typing****

For pooled sperm typing, we used a dilution of approximately 0.25 recombinant molecules per reaction. Based on the Poisson distribution, as described in ([Ng et al., 2008](#_ENREF_70)), this resulted in 78% reactions containing 0 recombinants (negative PCR) and 19% reactions containing 1 or ~2% containing more than one recombinants (positive reactions). These low dilution levels ensured that only ~2% of the reactions contained more than one recombinant molecules. In order to collect >4,400 single recombinant molecules, we required 4 times as many reactions. In the rare cases that the final genotyping rendered a heterozygous state for the internal SNPs (not possible when starting with one recombinant molecule), we classified these reactions as starting with more than one recombinant molecule and they were excluded from the analysis. However, we cannot exclude that these molecules were single recombinants with heteroduplex DNA.

For the selective amplification of COs, NCOs and non-recombinant molecules via pooled sperm typing, the primers for the flanking SNPs **(**rs7201177 C/G, rs1861187 C/T, rs4786854 C/T and rs4786855 A/C) were combined depending on the allelic phase of the respective haplotypes as shown in SM-Table S1. **The first PCR was conducted as a non-real-time PCR (non-rtPCR) in a 384-well plate format using 10 µl reaction volumes. For the 2^nd^ PCR, the 1:10 diluted product of the 1st PCR was used as template in a rtPCR reaction. Given the** perfect match of allele-specific primers at the 3’ end, and therefore a more specific and efficient amplification, reactions with one or more recombinant molecules rendered lower Cq values (earlier measureable amplification) in the 2^nd^ PCR than reactions without a recombinant molecule (non-recombinants).

In case of the non-crossover collection only the 1^st^ PCR was performed. The selection during the 2^nd^ PCR was performed by internal SNP genotyping reactions.

***PCR primers:***

| **PCR step** | **SNP ID** | **Primer** | **Primer ID** | **Sequence 5´- 3´** | **Amplicon length [bp]** |
| --- | --- | --- | --- | --- | --- |
| 1^st^ PCR | rs7201177 C/G | Forward | F-7201177(C) | TAG GAC GTC TCT CTG C*T*T*C (19) | 3390 bp |
|  |  |  | F-7201177(G) | TAG GAC GTC TCT CTG C*T*T*G (19) |  |
|  | rs4786855 A/C | Reverse | R-4786855(A) | GAA GTA GCA ATG AGA GAG AGA AG*A*A*T (26) |  |
|  |  |  | R-4786855(C) | GAA GTA GCA ATG AGA GAG AGA AG*A*A*G (26) |  |
| 2^nd^ PCR | rs1861187 C/T | Forward | F-1861187(C) | GCG ATT GAA ATA ATC AGG TC*T*C*A*C (24) | 3205 bp |
|  |  |  | F-1861187(T) | GCG ATT GAA ATA ATC AGG TC*T*C*A*T (24) |  |
|  | rs4786854 C/T | Reverse | R-4786854(C) | CCT TGT TTC CTG ATT ACA C A*G*C*G (23) |  |
|  |  |  | R-4786854(T) | CCT TGT TTC CTG ATT ACA C A*G*C*A (23) |  |

***1^st^ PCR conditions (non-real time PCR):***

| **Step** | **Temp.** | **Time** |  |
| --- | --- | --- | --- |
| **1** | **94°C** | 2 min |  |
| **2** | **94°C** | 15 sec | Go to step 2 for 5x |
| **3** | **65°C** | 15 sec |  |
| **4** | **72°C** | 1 min |  |
| **5** | **94°C** | 15 sec | Go to step 5 for 25x |
| **6** | **63** | 15 sec |  |
| **7** | **72** | 1:30min |  |
| **8** | **72°C** | 2min |  |
| **9** | **8°C** |  |  |

***2^nd^ PCR conditions (real time PCR):***

| **Step** | **Temp.** | **Time** |  |
| --- | --- | --- | --- |

| **1** | **94°C** | 2min |  |
| --- | --- | --- | --- |

| **2** | **94°C** | 15 sec | Go to step 2 for 45x |
| --- | --- | --- | --- |

| **3** | **65°C** | 15 sec |  |
| --- | --- | --- | --- |
| **4** | **72°C** | 1 min (plate read) |  |
| **5** | **82°C** | 5 sec (plate read) |  |
| **6** | **72°C** | 2 min |  |
| **7** | **65-95°C** | 5 sec / 0.5°C | Melt curve analysis |

#

# ****PCR conditions**** ****for CO internal SNP genotyping with TaqMan****

CO genotyping was performed via TaqMan® primer-probe combinations as published in ([Heissl et al., 2017](#_ENREF_37)). The primers (Eurofins) were universal and did not discriminate between alleles. All reactions were performed with Hot Taq DNA polymerase (peqlab). The numbers in brackets after the primer/probe sequence represent the oligo length.

***PCR primers:***

The allele-specific base of the probes (Eurofins) is shown in green bold and underlined and placed in the middle of the probe. Some probes are designed with phosphorothioate (PTO; *) to increase their specificity. FAM or HEX were chosen as 5´ fluorescent dyes and BHQ1 as 3´ quencher, which show overlapping absorption and emission spectra for optimal fluorescence resonance energy transfer (FRET).

| **SNP ID** | | **SNP position**  **(GRCh37/hg19)** | **Primer ID** | **Sequence 5´- 3´** | **Amplicon length [bp]** | **Annealing temp [°C]** |
| --- | --- | --- | --- | --- | --- | --- |
| rs12102448 A/G | | 6,360,774 | OF-12102448 | GTC AAA CTG TAC TGT CAC (18) | 143 | 60°C |
|  |  |  | 5´HEX/3´BHQ1/448(G)-Sense | CAG ATG TCT AC**G** AAT GAA GAG T (22) |  |  |
|  |  |  | 5´FAM/3´BHQ1/448(A)-Sense | CAG ATG TCT AC**A** AAT GAA GAG TC (23) |  |  |
|  |  |  | OR-12102448 | CAC TCT TAG AAT CCA GTT AG (20) |  |  |
| rs112051149 C/T | | 6,361,237 | OF-112051149 | AGA ATC TCT TGA ACA CAG GAG (21) | 85 | 61°C |
|  |  |  | 5´FAM/3´BHQ1/149(C)-Antis. | C AGC TCA *C*T***G** *C*AA CCT CTG (19) |  |  |
|  |  |  | 5´HEX/3´BHQ1/149(T)-Antis. | TCA GCT CA*C *T***A***C *AAC CTC TG (20) |  |  |
|  |  |  | OR-112051149 | GAG TCT CGT ACT GTC ACC (18) |  |  |
| rs72778219 C/T | | 6,361,632 | OF-72778219 | AGC AAC CAC AGG TTT ACA G (19) | 108 | 57°C |
|  |  |  | 5´FAM/3´BHQ1/219(C)-Sense | AGG CTG GA*G ***C***TC AG*G GTG (18) |  |  |
|  |  |  | 5´HEX/3´BHQ1/219(T)-Sense | AGG C*TG GA*G ***T***TC AG*G GTG C (19) |  |  |
|  |  |  | OR-72778219 | GTC TGC CAT GCG AAA GAT (18) |  |  |
| rs8060928 C/T | 6,361,951 | OF-8060928/2 | CTA ACC TCT CTA CCA CC (17) | 130 | 57°C |  |
|  |  | 5´FAM/3´BHQ1/928(C)-Antis. | TGT CCT TG*A ***G***AG GA*C CCT (18) |  |  |  |
|  |  | 5´HEX/3´BHQ1/928(T)-Antis. | TGT C*CT TG*A ***A***AG GA*C CCT (18) |  |  |  |
|  |  | OR-8060928/2 | TGA CCT CAT TCA GGT GTC (18) |  |  |  |

***PCR conditions:***

| **Step** | **Temp.** | **Time** |  |
| --- | --- | --- | --- |
| **1** | **95°C** | 2 min |  |
| **2** | **95°C** | 15 sec | Go to step 2 for 35x |
| **3** | **Probe annealing temp.°C** | 5 sec (plate read FAM/HEX) |  |
| **4**  **5** | **72°C**  **25°C** | 5 min  hold |  |

# ****PCR conditions for CO and NCO internal SNP genotyping with allele-specific primers****

For the microsatellites rs35094442 6A/7A, rs200121160 9A/19A, and SNP rs12102452 A/C a TaqMan® probe design was not possible. Therefore, allele-specific primers were designed as described in ([Heissl et al., 2017](#_ENREF_37)). COs were genotyped with an allele-specific assay only for the three above mentioned positions. NCO genotyping was exclusively performed with allele-specific PCR, using the primer for the converted allele at each SNP position. The reactions with conversion events were identified by lower Cq values than reactions with only non-recombinants.

***PCR primers:***

Allele-specific bases are shown in green, bold and underlined and mismatches in red. The last three 3´bases are synthesized with PTO bonds (*) to increase the specificity of the primers.

| **SNP ID** | **SNP position**  **(GRCh37/hg19)** | **Primer ID** | **Sequence 5´- 3´** | **Amplicon length [bp]** | **Annealing temp [°C]** | **Polymerase** |
| --- | --- | --- | --- | --- | --- | --- |
| rs12446415  A/G | 6,360,449 | F-12446415(A) | GTT GTT GAA GAA GTA G*C*C* **A** (19) | 90 | 56°C | iTaq (BioRad) |
|  |  | F-12446415(G) | GTT GTT GAA GAA GTA G*C*C* **G** (19) |  |  |  |
|  |  | OR-12446415 | GTG AGG ACA CTA CAG CTA (18) |  |  |  |
| rs11077029  C/T | 6,360,562 | F-35272019(C) | GGC ATT ATT ATA CCC ATT C*C*A* **C** (22) | 169 | 57°C | iTaq (BioRad) |
|  |  | F-35272019(T) | GGC ATT ATT ATA CCC ATT C*C*A* **T** (22) |  |  |  |
|  |  | OR-35272019 | GCT GTG CCC AGT CAT ATT CA (20) |  |  |  |
| rs35094442  6A/7A | 6,360,567 | OF-35094442 | GCT GTA GTG TCC TCA CAT CAA CCC (24) | 82 | 60°C | Phusion HSII Thermo Fisher Scientific) or iTaq (BioRad) |
|  |  | R-3509442(7A) | CCG CTT GGA GCT TCA GTT TT*G*T***T** (23) |  |  |  |
|  |  | R-3509442(6A) | CCG CTT GGA GCT TCA GTT TT*G*T***G** (23) |  |  |  |
| rs35272019  A/G | 6,360,766 | F-35272019(A) | AAG ACA GAT GTA GAG AC*A*G***A** (20) | 127 | 56°C | iTaq (BioRad) |
|  |  | F-35272019(G) | AAG ACA GAT GTA GAG AC*A*G***G** (20) |  |  |  |
|  |  | OR-35272019 | CAT AGC TTC AGG GAG TCC (18) |  |  |  |
| rs12102448  A/G | 6,360,774 | OF-12102448 | CCA AAA CCT GTA GGA TGT CA (20) | 150 | 62°C | iTaq (BioRad) |
|  |  | R-12102448(A) | GAG CAT CTC TAT AGA CTC TTC A*T*T***T** (25) |  |  |  |
|  |  | R-12102448(G) | GAG CAT CTC TAT AGA CTC TTC A*T*T***C** (25) |  |  |  |
| rs12102452  A/C | 6,360,887 | OF-12102452 | TAG AGA TGC TAA CTG GAT TCT AAG (18) | 121 | 52°C | Phusion HSII Thermo Fisher Scientific) iTaq (BioRad) |
|  |  | R-12102452(A) | CTG TTG AAA CCA CTG GTC A*G*T ***T** (22) |  |  |  |
|  |  | R-12102452(C) | TGA AAC CAC TGG TCA *G*T***G** (18) |  |  |  |
| rs200121160  9A/19A (1) | 6,360,903 | F-160(A19) | GCC GCA CAT TTA CCA GTG GTT TAA AAA ATA AA*A *A***A** (35) | 107 | 61°C | Phusion HSII (Thermo Fisher Scientific) |
|  |  | F-160(A9) | GCC CAT TTA CCA GTG GTT TAT AAT AA*A* A***G** (29) | 94 |  |  |
|  |  | OR-160 | TGT CCT AGC ATC TCT GAT AAC (21) |  |  |  |
| rs200121160  9A/19A (2) | 6,360,903 | F-160(A19) | GCA CAT TTA CCA GTG GTT TAA AAA ATA AA*A *A***A** (32) | 104 | 61°C | Phusion HSII (Thermo Fisher Scientific) |
|  |  | F-160(A9) | GCC CAT TTA CCA GTG GTT TAT AAT AA*A* A***G** (29) | 94 |  |  |
|  |  | OR-160 | TGT CCT AGC ATC TCT GAT AAC (21) |  |  |  |
| rs112051149  C/T | 6,361,237 | OF-112051149 | GGT CAG GAG TTC AAG ACC AGC (21) | 175 | 58°C | iTaq (BioRad) |
|  |  | R-112051149(C) | GGC ACG ATC TCA GCT CA*T *T***G** (20) |  |  |  |
|  |  | R-112051149(T) | GGC ACG ATC TCA GCT CA*T *T***A** (20) |  |  |  |
| rs72778219  C/T | 6,361,632 | F-72778219 (C) | ATT GCT TCT GAG GCT GG*C *G***C** (20) | 141 | 58°C | iTaq (BioRad) |
|  |  | F-72778219 (T) | ATT GCT TCT GAG GCT GG*C *G***T** (20) |  |  |  |
|  |  | OR-72778219 | CCA CAT AAC AGG AAG CTG CTT AG (23) |  |  |  |
| rs8060928  C/T | 6,361,951 | OF-8060928 | CGC ACA TCA TAA TCC CTG AGT G (22) | 92 | 60°C | iTaq (BioRad) |
|  |  | R-8060928(C) | GTC TGG CGT TTA ACT GTC CTT *G*A***G** (24) |  |  |  |
|  |  | R-8060928(T) | GTC TGG CGT TTA ACT GTC CTT *G*A***A** (24) |  |  |  |

*PCR condition iTaq polymerase (BioRad):*

| **Step** | **Temp.** | **Time** |  |
| --- | --- | --- | --- |
| **1** | **95°C** | 3 min |  |
| **2** | **95°C** | 15 sec | Go to step 2 for 35x |
| **3** | **Primer annealing temp.** | 5 sec |  |
| **4** | **72°C** | 15 sec |  |
| **5** | **72°C** | 7 min |  |
| **6** | **65-95°C** | 5 sec / 0.5°C | Melt curve analysis |

*PCR condition Phusion HSII (Thermo Fisher Scientific) for rs35094442 6A/7A and rs12102452 A/C:*

| **Step** | **Temp.** | **Time** |  |
| --- | --- | --- | --- |
| **1** | **94°C** | 2 min |  |
| **2** | **94°C** | 15 sec | Go to step 2 for 45x |
| **3** | **Primer annealing temp.** | 5 sec |  |
| **4** | **72°C** | 10 sec |  |
| **5** | **72°C** | 7 min |  |
| **6** | **65-95°C** | 5 sec / 0.5°C | Melt curve analysis |

*PCR condition 1 Phusion HSII (Thermo Fisher Scientific) for rs200121160 19A/9A:*

| **Step** | **Temp.** | **Time** |  |
| --- | --- | --- | --- |
| **1** | **94°C** | 2 min |  |
| **2** | **94°C** | 15 sec | Go to step 2 for 5x |
| **3** | **56°C** | 5 sec |  |
| **4** | **63°C** | 15 sec |  |
| **5** | **94°C** | 15 sec | Go to step 2 for 35x |
| **6** | **53°C** | 5 sec |  |
| **7** | **58°C** | 15 sec |  |
| **8** | **58°C** | 30 min |  |
| **9** | **65-95°C** | 5 sec / 0.5°C | Melt curve analysis |

*PCR condition 2 Phusion HSII (Thermo Fisher Scientific) for rs200121160 19A/9A*

These conditions were used only for donor 1391 CO and NCO genotyping.

| **Step** | **Temp.** | **Time** |  |
| --- | --- | --- | --- |
| **1** | **94°C** | 2 min |  |
| **2** | **94°C** | 15 sec | Go to step 2 for 45x |
| **3** | **56°C** | 5 sec |  |
| **4** | **68°C** | 15 sec |  |
| **5** | **68°C** | 30 min |  |
| **6** | **65-95°C** | 5 sec / 0.5°C | Melt curve analysis |

# Testing the number of amplifiable genomes

To estimate the number of amplifiable genomes for each donor and experiment, we followed the same approach as described in ([Arbeithuber et al., 2015](#_ENREF_4)). In short, non-recombinant amplifiable DNA was assessed with pooled sperm typing using 0.8-2.0 genomes of sperm DNA per reaction and 4.0 – 7.0 pg of *E.Coli* DNA as a carrier DNA (SM-Table S2). We measured ~20% positive reactions, with 78% reactions containing 0 molecules, 19% reactions containing 1 molecule and 2% of reactions containing 2 molecules estimated from the Poisson distribution ([Ng et al., 2008](#_ENREF_70)). An example of how the number of meiotic molecules (amplifiable sperm) were then estimated is shown below.

**E.g. Donor 1027:** In total, 177 reactions containing ~2 sperm genomes per reaction (as determined spectrophotometrically) and 800 genomes (4 pg) of *E.Coli* DNA were amplified with the same conditions as in pooled sperm typing using primers targeting the non-recombinant phase, resulting in 177*2 = 354 . Since only 42 of 177 reactions rendered an early amplification, we calculated 42/177= 23.73% positives and 100-23.73= 76.27% negatives. This number was then adjusted to the Poisson distribution, resulting in (-LN(0.7627))=0.27 observed molecules per reaction. Thus, the effective number of positive reactions was 177*0.27= 47.9. The correction factor was then calculated by dividing the effective positive reactions by the number of meiosis equal to the sperm genomes (47.9/354=0.14). The amplifiable sperm for a CO or NCO were estimated by the number of meiosis (sperm genomes determined by the OD) used in each experiment multiplied by the correction factor. This was performed for each donor and recombinant type with these additional control experiments.

|  |  |  |  | *Step 1* | *Step 2* | *Step 3* | *Step 4* | *Step 5* |
| --- | --- | --- | --- | --- | --- | --- | --- | --- |
| **Copies/**  **reaction** | **Total # of reactions** | **Sperms** | **Positives** | **Negatives**  **(1-positives)** | **Observed**  **molecules/reaction**  **(-Ln(negatives))** | **Effective positive**  **reactions** | **Correction factor** | **Amplifiable sperm used for CO collection**  **(sperm * correction f.)** |
| 2.0 | 177 | 354 | 44 (23.73%) | 76.27% | 0.27 | 177*0.27=47.9 | 47.9/354 = 0.14 | 3,964,971*0.14=555,096 |

# Data analysis

## Poisson correction of CO events

Collected COs or NCOs per donor and reciprocal were estimated based on the number of positive reactions corrected by the Poisson distribution as described below:

**E.g. Donor 1100 RI:** In total, 1,673 pooled sperm typing reactions containing each ~4 ng of sperm DNA equivalent to 1,200 genomes or 1,673*1,200 = 2,007,600 sperms resulted in 298 positives or 17.8% (298/1673) positives with one or more CO. The percentage of negatives is 82.2% (100-17.8) used to infer the Poisson estimate of single CO/reaction of 20% (-LN(0.822)), or in total 334.6 CO (1,673 * 0.20).

|  |  |  |  | *Step 1* | *Step 2* | *Step 5* |
| --- | --- | --- | --- | --- | --- | --- |
| **Copies/**  **reaction** | **Total reactions** | **Sperms** | **Positives** | **Negatives**  **(1-positives)** | **Observed**  **CO/reaction**  **(-Ln(negatives))** | **Poisson corrected CO** |
| 1,200 | 1,673 | 2,007,600 | 298 (17.8%) | 82.2% | 0.20 | 1,673 * 0.20= **334.6** |

## Calculation of recombination frequencies

The CO frequency (CO events per number of meiosis) were calculated by using the Poisson corrected number of COs measured for R1 or R2 divided by a fourth of the number of amplifiable sperm (equation 1), since 4 sperm result from one meiosis.

(1)


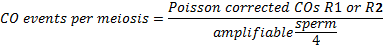

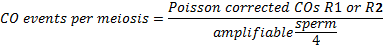


The NCO frequencies (NCO events per meiosis) were calculated by dividing the Poisson corrected number of NCOs by a fourth of the number of amplifiable sperm (equation 2), since 4 sperm result from one meiosis.

(2)


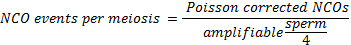

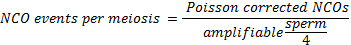


## Significance testing of recombination frequencies

Significance tests of recombination frequencies were performed in R with an exact test of a simple null hypothesis for the rate parameter in Poisson distribution ([Fay, 2010](#_ENREF_29)).

## Calculation of CO and NCO centers

CO and NCO centers were calculated by fitting the cumulative CO or NCO frequencies (calculated as described in SM-Material and Methods 8b) and plotted against the chromosome position in OriginPro 2018G. For COs the middle position between two heterozygous SNPs, where the recombination occurred, was used as “chromosome position”. For estimating NCOs centers, we used directly the SNP where the conversion occurred.

CO centers were inferred by fitting the data points with a normal cumulative distribution function (y0= offset, A=amplitude, xc= mean, w= standard deviation; equation 3) with xc representing the CO center.

(3)


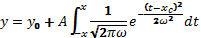

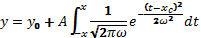


NCO centers were calculated by fitting the data points with a sigmoidial logistic function type 1 (k=steepness, a=curve maximum; equation 4) with xc representing the NCO center.

(4)


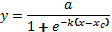

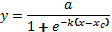


## Log rate ratio plots

Log rate ratio plots were calculated to analyze transmission biases (equation 5).


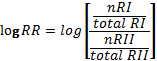


logRR=0 denotes the expected equal transmission of alleles between the reciprocal recombinant haplotypes. LogRR>0 represents the over-transmission of RI and logRR<0 the over-transmission of RI. Significant tests were performed after standardized Pearson residual.

## Poly-A transmission rate calculation

**from Table S2:**

The CO rate is 4.98 x 10^-3^

NCO ratio is 2.83x 10^-3^

Thus, the proportion of CO+NCO molecules that are

CO = 0.637644

and

NCO = 1-0.637644 =0.362356

**from Table 1**

- **For 6A/7A:**

58.0% of CO

and

55.4% of NCO

inherit the 7A allele.

Averaged over both, that means 0.637644 * 0.58 + 0.362356 * 0.554 = 57.1% of CO and NCO molecules inherit the 7A allele. (Presumably the others are 50/50.)

- **For 9A/19A:**

50.2% of CO

and

0.0% of NCO

inherit the 19A allele

Averaged over both, that means 0.637644 * 0.502 + 0.362356 * 0.0= 32% of CO and NCO molecules inherit the 19A allele.

References SM

ALLERS, T. & LICHTEN, M. 2001. Differential timing and control of noncrossover and crossover recombination during meiosis. *Cell,* 106, 47-57.

ALTEMOSE, N., NOOR, N., BITOUN, E., TUMIAN, A., IMBEAULT, M., CHAPMAN, J. R., ARICESCU, A. R. & MYERS, S. R. 2017. A map of human PRDM9 binding provides evidence for novel behaviors of PRDM9 and other zinc-finger proteins in meiosis. *Elife,* 6.

ARBEITHUBER, B., BETANCOURT, A. J., EBNER, T. & TIEMANN-BOEGE, I. 2015. Crossovers are associated with mutation and biased gene conversion at recombination hotspots. *Proc Natl Acad Sci U S A,* 112, 2109-14.

ARBEITHUBER, B., HEISSL, A. & TIEMANN-BOEGE, I. 2017. Haplotyping of Heterozygous SNPs in Genomic DNA Using Long-Range PCR. *Methods Mol Biol,* 1551, 3-22.

BERG, I. L., NEUMANN, R., LAM, K. W., SARBAJNA, S., ODENTHAL-HESSE, L., MAY, C. A. & JEFFREYS, A. J. 2010. PRDM9 variation strongly influences recombination hot-spot activity and meiotic instability in humans. *Nat Genet,* 42, 859-63.

FAY, M. P. 2010. Two-sided Exact Tests and Matching Confidence Intervals for Discrete Data *The R Journal,* 2, 53-58.

FUNGTAMMASAN, A., ANANDA, G., HILE, S. E., SU, M. S. W., SUN, C., HARRIS, R., MEDVEDEV, P., ECKERT, K. & MAKOVA, K. D. 2015. Accurate typing of short tandem repeats from genome-wide sequencing data and its applications. *Genome Research,* 125, 736-749.

HEISSL, A., ARBEITHUBER, B. & TIEMANN-BOEGE, I. 2017. High-Throughput Genotyping with TaqMan Allelic Discrimination and Allele-Specific Genotyping Assays. *Methods Mol Biol,* 1492, 29-57.

NG, S. H., PARVANOV, E., PETKOV, P. M. & PAIGEN, K. 2008. A quantitative assay for crossover and noncrossover molecular events at individual recombination hotspots in both male and female gametes. *Genomics,* 92, 204-9.

PATEL, A., ZHANG, X., BLUMENTHAL, R. M. & CHENG, X. 2017. Structural basis of human PR/SET domain 9 (PRDM9) allele C-specific recognition of its cognate DNA sequence. *J Biol Chem,* 292, 15994-16002.

PRATTO, F., BRICK, K., KHIL, P., SMAGULOVA, F., PETUKHOVA, G. V. & CAMERINI-OTERO, R. D. 2014. DNA recombination. Recombination initiation maps of individual human genomes. *Science,* 346, 1256442.
