# Supplementary material for: The impact of poly-A microsatellite heterologies in meiotic recombination
Source: Life Sci Alliance. 2019 Apr 25;2(2):e201900364. doi: 10.26508/lsa.201900364 (PMC6485458; doi:10.26508/lsa.201900364)
Supplement: Supplementary file 2 [file LSA-2019-00364_TableS1.docx]

Supplementary Tables

**Supplement Table S1. Donor genotypes and haplotypes**

Haplotypes and genotypes of each parental allele (NRI or NRII) of the eight informative donors are shown. The table includes the flanking SNPs targeted for the CO and NCO collection (first and last two rows), as well as, the informative SNPs within the hotspot together with their chromosomal positions (hg19, chr.16), and allelic state. Heterozygote sites within the hotspot are marked in red and blue.

**9A/19A Donors:**

| **SNP** | | **Position** | **Donor 1027** | | **Donor 1034** | | **Donor 1081** | | **Donor 1391** | | |
| --- | --- | --- | --- | --- | --- | --- | --- | --- | --- | --- | --- |
|  |  |  | **NRI**  **GCCC** | **NRII**  **CTTA** | **NRI**  **GCCC** | **NRII**  **CTTA** | **NRI**  **GCCC** | **NRII**  **CTTA** | **NRI**  **GCTA** | **NRII**  **CTCC** |  |
| rs7201177 | C/G | 6358968 | G | C | G | C | G | C | G | C |  |
| rs1861187 | C/T | 6359077 | C | T | C | T | C | T | C | T |  |
| rs12446415 | A/G | 6360449 | G | G | G | G | G | G | A | G |  |
| rs11077029 | C/T | 6360562 | T | T | T | T | T | T | C | T |  |
| rs35094442 | 6A/7A | 6360567 | 6A | 7A | 6A | 7A | 6A | 7A | 7A | 7A |  |
| rs35272019 | A/G | 6360766 | A | A | A | A | A | A | G | A |  |
| rs12102448 | A/G | 6360774 | A | G | A | G | A | G | G | G |  |
| rs12102452 | A/C | 6360887 | A | C | A | C | A | C | C | C |  |
| rs200121160 | 9A/19A | 6360903 | 19A | 9A | 19A | 9A | 19A | 9A | 19A | 9A |  |
| rs112051149 | C/T | 6361237 | C | C | C | T | C | C | T | C |  |
| rs72778219 | C/T | 6361632 | T | C | T | C | T | T | C | T |  |
| rs12445929 | C/T | 6361908 | T | C | T | C | T | C | C | T |  |
| rs8060928 | C/T | 6361951 | T | C | T | C | T | C | C | T |  |
| rs4786854 | C/T | 6362280 | C | T | C | T | C | T | T | C |  |
| rs4786855 | A/C | 6362356 | C | A | C | A | C | A | A | C |  |

**19A/19A Donors:**

| **SNP** | | **Position** | **Donor 1100** | | **Donor 1227** | | **Donor 1251** | | **Donor 1288** | | |
| --- | --- | --- | --- | --- | --- | --- | --- | --- | --- | --- | --- |
|  |  |  | **NRI**  **GCCC** | **NRII**  **CTTA** | **NRI**  **GCCC** | **NRII**  **CTTA** | **NRI**  **GCTA** | **NRII**  **CTCC** | **NRI**  **GCCC** | **NRII**  **CTTA** |  |
| rs7201177 | C/G | 6358968 | G | C | G | C | G | C | G | C |  |
| rs1861187 | C/T | 6359077 | C | T | C | T | C | T | C | T |  |
| rs12446415 | A/G | 6360449 | G | G | G | G | A | A | G | G |  |
| rs11077029 | C/T | 6360562 | T | T | T | T | C | C | T | T |  |
| rs35094442 | 6A/7A | 6360567 | 6A | 7A | 6A | 7A | 7A | 6A | 6A | 7A |  |
| rs35272019 | A/G | 6360766 | A | A | A | A | G | G | A | A |  |
| rs12102448 | A/G | 6360774 | A | G | G | G | G | A | A | G |  |
| rs12102452 | A/C | 6360887 | A | A | A | C | C | A | C | A |  |
| rs200121160 | 9A/19A | 6360903 | 19A | 19A | 19A | 19A | 19A | 19A | 19A | 19A |  |
| rs112051149 | C/T | 6361237 | C | T | C | T | T | C | C | C |  |
| rs72778219 | C/T | 6361632 | T | C | T | C | C | T | T | C |  |
| rs12445929 | C/T | 6361908 | T | C | T | C | C | T | T | C |  |
| rs8060928 | C/T | 6361951 | T | C | T | C | C | T | T | C |  |
| rs4786854 | C/T | 6362280 | C | T | C | T | T | C | C | T |  |
| rs4786855 | A/C | 6362356 | C | A | C | A | A | C | C | A |  |
