# Supplementary material for: The impact of poly-A microsatellite heterologies in meiotic recombination
Source: Life Sci Alliance. 2019 Apr 25;2(2):e201900364. doi: 10.26508/lsa.201900364 (PMC6485458; doi:10.26508/lsa.201900364)
Supplement: Supplementary file 3 [file LSA-2019-00364_TableS2.docx]

**Supplement Table S2. Crossover and non-crossover frequency estimation.**

The recombination frequency was estimated as the ratio of CO or NCO (Poisson corrected) per meiosis. Note that only one of the CO reciprocals can be captured per reaction with pooled sperm typing, so the CO number per meiosis was estimated as the Poisson corrected COs divided by a fourth of the number of amplifiable sperm given that each meiosis produces four sperm (e.g. 1027: 901/(555,096/4) = 6.5 x 10^-3^ for the CO frequency). The same applies for NCOs since for NCO we assume that ~99% come from unidirectional SDSA repair and are the product of one meiotic division ([Allers and Lichten, 2001](#_ENREF_1)). For details also see SM –section 8. The number of amplifiable sperm was determined using the average concentration of sperm (determined spectrophotometrically) multiplied by a correction factor derived from non-recombinant controls (Methods and Materials and SM Table 11 and SM Materials and Methods-Point 7). Correction factors across experiments were within 0.10-0.49 differing between individual donors. Collected recombinant molecules were Poisson corrected (Supplement methods and materials section 7 and 8 for more details).

| **Group** | **Donor ID** | **Poisson**  **corrected**  **COs** | **Amplifiable sperm** | **sperm molecules**  **per reaction** | **CO frequency (*10^-3^)** | **95% Poisson CI lower** | **95% Poisson CI upper** | **Poisson corrected**  **NCOs** | **Amplifiable**  **sperm** | **sperm**  **molecules**  **per reaction** | **NCO frequency (*10^-3^)** | **95% Poisson CI lower** | **95% Poisson CI upper** |
| --- | --- | --- | --- | --- | --- | --- | --- | --- | --- | --- | --- | --- | --- |
| 9A/19A | 1027 | 901 | 555,096 | 800 and 900 | 6.49 | 6.08 | 6.93 | 63 | 30,250 | 500 | 8.33 | 6.40 | 10.66 |
|  | 1034 | 1,466 | 1,469,600 | 1000 | 3.99 | 3.79 | 4.20 | 37 | 27,414 | 500 | 5.40 | 3.80 | 7.44 |
|  | 1081 | 582 | 776,355 | 600 | 3.00 | 2.76 | 3.25 | 17 | 33,000 | 500 | 2.06 | 1.20 | 3.30 |
|  | 1391 | 151 | 131,320 | 1400 | 4.60 | 3.90 | 5.39 | 21 | 34,320 | 500 | 2.45 | 1.52 | 3.74 |
| 19A/19A | 1100 | 656 | 364,800 | 600 | 7.19 | 6.65 | 7.77 | 26 | 96,250 | 500 | 1.08 | 0.71 | 1.58 |
|  | 1227 | 390 | 213,920 | 800 | 7.29 | 6.59 | 8.05 | 13 | 55,000 | 500 | 0.95 | 0.50 | 1.62 |
|  | 1251 | 298 | 182,640 | 1200 | 6.53 | 5.81 | 7.31 | 52 | 53,040 | 500 | 3.92 | 2.93 | 5.14 |
|  | 1288 | 476 | 254,688 | 800 | 7.48 | 6.82 | 8.18 | 26 | 31,200 | 500 | 3.33 | 2.18 | 4.88 |
| **9A/19A** |  | 3,100 | 2,932,370 |  | 4.23 | 4.08 | 4.38 | 138 | 124,984 |  | 4.42 | 3.71 | 5.22 |
| **19A/19A** |  | 1,820 | 1,016,048 |  | 7.17 | 6.84 | 7.51 | 117 | 235,490 |  | 1.99 | 1.64 | 2.38 |
| **Sum** |  | **4,920** | **3,948,418** |  | **4.98** | **4.85** | **5.13** | **255** | **360,474** |  | **2.83** | **2.49** | **3.20** |
