# Supplementary material for: The impact of poly-A microsatellite heterologies in meiotic recombination
Source: Life Sci Alliance. 2019 Apr 25;2(2):e201900364. doi: 10.26508/lsa.201900364 (PMC6485458; doi:10.26508/lsa.201900364)
Supplement: Supplementary file 4 [file LSA-2019-00364_TableS3.docx]

**Supplement Table S3. CO per reciprocal**

CO frequencies were calculated per individual reciprocal by considering the number of collected recombinants and dividing by a fourth of amplifiable meiosis (e.g. for donor 1027 we estimated 475.2/(276,520/4)= 6.87 x 10^-3^). Collected numbers of CO were Poisson corrected (SM-Material and Methods section 8). Both reciprocals show equal CO frequencies for all donors and all donor groups. Note that the number of amplifiable sperm was divided by four since four sperm are produced per meiotic division. In the case of COs, only one product was assayed and it is equivalent to the number of meiosis resulting in a crossover.

| **Group** | **Donor ID** | **Poisson corrected COs** | | | | **Amplifiable sperm** | | | **c (*10^-3^)** | **95% Poisson CI** | | **c (*10^-3^)** | **95% Poisson CI** | | **c (*10^-3^)** | **95% Poisson CI** | | |
| --- | --- | --- | --- | --- | --- | --- | --- | --- | --- | --- | --- | --- | --- | --- | --- | --- | --- | --- |
|  |  | **Total** | **RI** | **RII** | **Total** | | **RI** | **RII** | **Total** | **lower** | **upper** | **RI** | **lower** | **upper** | **RII** | **lower** | **upper** |  |
| 9A/19A | 1027 | 901 | 475.2 | 425.6 | 555,096 | | 276,520 | 278,576 | 6.49 | 6.08 | 6.93 | 6.87 | 6.27 | 7.52 | 6.11 | 5.55 | 6.73 |  |
|  | 1034 | 1,466 | 749.2 | 716.5 | 1,469,600 | | 755,480 | 714,120 | 3.99 | 3.79 | 4.20 | 3.97 | 3.69 | 4.26 | 4.01 | 3.73 | 4.32 |  |
|  | 1081 | 582 | 302.0 | 280.0 | 776,354 | | 379,875 | 396,479 | 3.00 | 2.76 | 3.25 | 3.18 | 2.83 | 3.56 | 2.82 | 2.50 | 3.18 |  |
|  | 1391 | 151 | 81.1 | 69.8 | 131,320 | | 65,660 | 65,660 | 4.60 | 3.90 | 5.39 | 4.94 | 3.92 | 6.13 | 4.25 | 3.32 | 5.39 |  |
| 19A/19A | 1100 | 656 | 328.2 | 327.8 | 364,800 | | 190,722 | 174,078 | 7.19 | 6.65 | 7.77 | 6.88 | 6.15 | 7.67 | 7.53 | 6.74 | 8.40 |  |
|  | 1227 | 390 | 197.4 | 192.3 | 213,920 | | 107,100 | 106,820 | 7.29 | 6.59 | 8.05 | 7.37 | 6.37 | 8.46 | 7.20 | 6.21 | 8.28 |  |
|  | 1251 | 298 | 149.8 | 148.7 | 182,640 | | 91,560 | 91,080 | 6.53 | 5.81 | 7.31 | 6.54 | 5.55 | 7.69 | 6.53 | 5.54 | 7.68 |  |
|  | 1288 | 476 | 223.9 | 252.5 | 254,688 | | 127,008 | 127,680 | 7.48 | 6.82 | 8.18 | 7.05 | 6.16 | 8.04 | 7.91 | 6.98 | 8.97 |  |
| **9A/19A** |  | 3,100 | 1607.4 | 1491.9 | 2,932,370 | | 1,477,535 | 1,454,835 | 4.23 | 4.08 | 4.38 | 4.35 | 4.14 | 4.57 | 4.10 | 3.90 | 4.32 |  |
| **19A/19A** |  | 1,820 | 899.3 | 921.3 | 1,016,048 | | 516,390 | 499,658 | 7.17 | 6.84 | 7.51 | 6.97 | 6.52 | 7.43 | 7.38 | 6.90 | 7.86 |  |
| **Sum** |  | **4,920** | 2506.8 | 2413.2 | 3,948,418 | | 1,993,925 | 1,954,493 | 4.98 | 4.85 | 5.13 | 5.03 | 4.83 | 5.23 | 4.94 | 4.74 | 5.14 |  |
