# Supplementary material for: The impact of poly-A microsatellite heterologies in meiotic recombination
Source: Life Sci Alliance. 2019 Apr 25;2(2):e201900364. doi: 10.26508/lsa.201900364 (PMC6485458; doi:10.26508/lsa.201900364)
Supplement: Supplementary file 5 [file LSA-2019-00364_TableS4.docx]

**Supplement Table S4. NCOs per reciprocal**

NCO frequencies (meiosis resulting in NCO events) were calculated per reciprocal. The NCO frequency was estimated by dividing the number of Poisson corrected NCO events by one fourth of the number of amplifiable sperm (e.g. for donor 1027: 63/(30,250/4)=4.14 x 10^-3^). Same as for COs, the NCO frequencies were not significantly different between NRI and NRII. Collected NCOs were Poisson corrected (SM-Material and Methods 8). Note that only one fourth of amplifiable sperm was used, since four sperm are produced per meiotic division. We assumed that ~99% of NCOs resulted from the SDSA pathway ([Allers and Lichten, 2001](#_ENREF_1)), so that one NCOs represents a meiotic division.

| **Group** | **Donor ID** | **Poisson corrected NCOs** | | | **Amplifiable sperm** | | | **c (*10^-3^)** | **95% Poisson CI** | | **c (*10^-3^)** | **95% Poisson CI** | | **c (*10^-3^)** | **95% Poisson CI** | | |
| --- | --- | --- | --- | --- | --- | --- | --- | --- | --- | --- | --- | --- | --- | --- | --- | --- | --- |
|  |  | **Total** | **NRI** | **NRII** | **Total** | **NRI** | **NRII** | **Total** | **lower** | **upper** | **NRI** | **lower** | **upper** | **NRII** | **lower** | **upper** |  |
| 9A/19A | 1027 | 63 | 26.2 | 36.3 | 30,250 | 15,125 | 15,125 | 8.33 | 6.40 | 10.66 | 6.93 | 4.53 | 10.14 | 9.60 | 6.73 | 13.27 |  |
|  | 1034 | 37 | 19.7 | 17.5 | 27,414 | 13,707 | 13,707 | 5.40 | 3.80 | 7.44 | 5.75 | 3.50 | 8.91 | 5.11 | 3.00 | 8.12 |  |
|  | 1081 | 17 | 12.3 | 5.0 | 33,000 | 16,500 | 16,500 | 2.06 | 1.20 | 3.30 | 2.98 | 1.56 | 5.17 | 1.21 | 0.39 | 2.83 |  |
|  | 1391 | 21 | 12.2 | 9.1 | 34,320 | 17,160 | 17,160 | 2.45 | 1.52 | 3.74 | 2.84 | 1.48 | 4.95 | 2.12 | 0.97 | 4.01 |  |
| 19A/19A | 1100 | 26 | 21.8 | 4.0 | 96,250 | 48,125 | 48,125 | 1.08 | 0.71 | 1.58 | 1.81 | 1.13 | 2.75 | 0.33 | 0.09 | 0.85 |  |
|  | 1227 | 13 | 6.1 | 7.1 | 55,000 | 27,500 | 27,500 | 0.95 | 0.50 | 1.62 | 0.89 | 0.33 | 1.92 | 1.03 | 0.42 | 2.12 |  |
|  | 1251 | 52 | 18.5 | 33.8 | 53,040 | 26,520 | 26,520 | 3.92 | 2.93 | 5.14 | 2.79 | 1.67 | 4.38 | 5.10 | 3.53 | 7.13 |  |
|  | 1288 | 26 | 11.2 | 14.3 | 31,200 | 15,600 | 15,600 | 3.33 | 2.18 | 4.88 | 2.87 | 1.44 | 5.11 | 3.67 | 2.02 | 6.12 |  |
| **9A/19A** |  | 138 | 70.4 | 68.0 | 124,984 | 62,492 | 624,92 | 4.42 | 3.71 | 5.22 | 4.51 | 3.52 | 5.69 | 4.35 | 3.38 | 5.52 |  |
| **19A/19A** |  | 117 | 57.7 | 59.2 | 235,490 | 117,745 | 117,745 | 1.99 | 1.64 | 2.38 | 1.96 | 1.49 | 2.54 | 2.01 | 1.53 | 2.59 |  |
| **Sum** |  | 255 | 128.1 | 127.2 | 360,474 | 180,237 | 180,237 | 2.83 | 2.49 | 3.20 | 2.84 | 2.37 | 3.38 | 2.82 | 2.35 | 3.36 |  |
