# Supplementary material for: The impact of poly-A microsatellite heterologies in meiotic recombination
Source: Life Sci Alliance. 2019 Apr 25;2(2):e201900364. doi: 10.26508/lsa.201900364 (PMC6485458; doi:10.26508/lsa.201900364)
Supplement: Supplementary file 6 [file LSA-2019-00364_TableS5.docx]

**Supplement Table S5. Crossover and non-crossover centers.**

**CO and NCO centers were calculated as described in SM-Material and Methods section 8. CO centers were located upstream of NCO centers ranging between 40-330 bp within individual donors. The mean CO and NCO centers of all donors were separated by 80 bp, with the CO center located more upstream.**

| **State** | **Donor** | **CO center**  **RI [bp]** | **CO center**  **RII [bp]** | **CO center**  **RI+II [bp]** | **NCO center**  **NRI+II [bp]** | **Difference between CO and NCO center [bp]** |
| --- | --- | --- | --- | --- | --- | --- |
| 9A/19A | 1027 | 6,360,810 ± 26 | 6,360,870 ± 11 | 6,360,790 ± 20 | 6,360,890 ± 4 | -100 |
|  | 1034 | 6,360,880 ± 24 | 6,360,880 ± 24 | 6,360,880 ± 24 | 6,360,980 ± 73 | -100 |
|  | 1081 | 6,360,780 ± 16 | 6,360,770 ± 20 | 6,360,770 ± 10 | 6,360,890 ± 76 | -120 |
|  | 1391 | 6,360,760 ± 7 | 6,360,760 ± 13 | 6,360,760 ± 7 | 6,360,800 ± 75 | -40 |
| 19A/19A | 1100 | 6,360,860 ± 20 | 6,360,870 ± 27 | 6,360,860 ± 15 | 6,361,160 ± 245 | -300 |
|  | 1227 | 6,360,910 ± 19 | 6,360,860 ± 9 | 6,360,880 ± 23 | 6,360,910 ± 51 | -50 |
|  | 1251 | 6,360,970 ± 14 | 6,360,800 ± 26 | 6,360,890 ± 41 | 6,361,220 ± 244 | -330 |
|  | 1288 | 6,360,770 ± 1 | 6,360,760 ± 4 | 6,360,760 ± 14 | 6,360,920 ± 73 | -160 |
| 9A/19A |  |  |  | 6,360,770 ± 9 | 6,360,980 ± 71 | -210 |
| 19A/19A |  |  |  | 6,360,815 ± 19 | 6,360,830 ± 14 | -15 |
| Mean |  |  |  | 6,360,780 ± 9 | 6,360,860 ± 15 | -80 |
