# Supplementary material for: The impact of poly-A microsatellite heterologies in meiotic recombination
Source: Life Sci Alliance. 2019 Apr 25;2(2):e201900364. doi: 10.26508/lsa.201900364 (PMC6485458; doi:10.26508/lsa.201900364)
Supplement: Supplementary file 7 [file LSA-2019-00364_TableS6.docx]

**Supplement Table S6. NCOs: Simple- , co- and complex conversions**

We analyzed the collected NCOs for simple-, co- and complex conversion tracts by dividing the NCOs per type (simple, co-conversion or complex NCO) measured per donor by the total amount of collected NCOs for that donor (e.g. for donor 1027 simple conversions: 47/59 = 79,7%). While the proportion of complex NCOs/total NCO was nearly the same in Ht and Ho donors, there were slightly more simple conversions in Ho donors, but more co-conversions in Ht. We also observed a greater variance in conversion tract length for Ht donors (SM-Table S7).

| **Type** | **Donor** | **observed NCOs** | **Simple conversions** | | | | **Co-conversions** | | | | | | | | **Complex Conversions** | | | | | | | |
| --- | --- | --- | --- | --- | --- | --- | --- | --- | --- | --- | --- | --- | --- | --- | --- | --- | --- | --- | --- | --- | --- | --- |
|  |  |  | **1 SNP** | **[%]** | **95% Poisson CI** | | **2 SNPs** | **[%]** | **95% Poisson CI** | | **3 SNPs** | **[%]** | **95% Poisson CI** | | **1 SNP** | **[%]** | **95% Poisson CI** | | **2 SNPs** | **[%]** | **95% Poisson CI** | |
|  |  |  |  |  | **lower** | **upper** |  |  | **lower** | **upper** |  |  | **lower** | **upper** |  |  | **lower** | **upper** |  |  | **lower** | **upper** |
| 9A/19A | 1027 | 59 | 47 | 79,7 | 58.5 | 105.9 | 1 | 1.7 | 0.0 | 9.4 | 1 | 1.7 | 0.0 | 9.4 | 10 | 16.9 | 8.1 | 31.2 |  |  |  |  |
|  | 1034 | 36 | 31 | 86.1 | 58.5 | 122.2 | 1 | 2.8 | 0.1 | 15.5 | 1 | 2.8 | 0.1 | 15.5 | 1 | 2.8 | 0.1 | 15.5 | 2 | 5.6 | 0.7 | 20.1 |
|  | 1081 | 17 | 12 | 70.6 | 36.5 | 123.3 | 5 | 29.4 | 9.5 | 68.6 |  |  |  |  |  |  |  |  |  |  |  |  |
|  | 1391 | 21 | 15 | 71.4 | 40.0 | 117.8 |  |  |  |  |  |  |  |  | 6 | 28.6 | 10.5 | 62.2 |  |  |  |  |
| 19A/19A | 1100 | 25 | 22 | 88.0 | 55.1 | 133.2 | 1 | 4.0 | 0.1 | 22.3 |  |  |  |  | 2 | 8.0 | 1.0 | 28.9 |  |  |  |  |
|  | 1227 | 13 | 10 | 76.9 | 36.9 | 141.5 | 1 | 7.7 | 0.2 | 42.9 |  |  |  |  | 2 | 15.4 | 1.9 | 55.6 |  |  |  |  |
|  | 1251 | 50 | 41 | 82.0 | 58.8 | 111.2 |  |  |  |  | 1 | 2.0 | 0.1 | 11.1 | 8 | 16.0 | 6.9 | 31.5 |  |  |  |  |
|  | 1288 | 25 | 25 | 100.0 | 64.7 | 147.6 |  |  |  |  |  |  |  |  |  |  |  |  |  |  |  |  |
| 9A/19A |  | 133 | 105 | 78.9 | 64.6 | 95.6 | 7 | 5.3 | 2.1 | 10.8 | 2 | 1.5 | 0.2 | 5.4 | 17 | 12.8 | 7.4 | 20.5 | 2 | 1.5 | 0.2 | 5.4 |
| 19A/19A |  | 113 | 98 | 86.7 | 70.4 | 105.7 | 2 | 1.8 | 0.2 | 6.4 | 1 | 0.9 | 0.0 | 4.9 | 12 | 10.6 | 5.5 | 18.6 |  |  |  |  |
| **Total** |  | **246** | **203** | **82.5** | **71.6** | **94.7** | **9** | **3.7** | **1.7** | **6.9** | **3** | **1.2** | **0.3** | **3.6** | **29** | **11.8** | **7.9** | **16.9** | **2** | **0.8** | **0.1** | **2.9** |
