# Supplementary material for: The impact of poly-A microsatellite heterologies in meiotic recombination
Source: Life Sci Alliance. 2019 Apr 25;2(2):e201900364. doi: 10.26508/lsa.201900364 (PMC6485458; doi:10.26508/lsa.201900364)
Supplement: Supplementary file 8 [file LSA-2019-00364_TableS7.docx]

**Supplement Table S7. Conversion tract length in non-crossover events**

Mean tract lengths of NCOs as well as co-conversion and complex conversion tract lengths are shown for each donor. The tract lengths were calculated with a weighted mean, starting and ending with SNPs flanking the conversion site (main manuscript Figure 3 panel D and E).

| **State** | **Donor** | **Mean tract length [bp]** | **Shortest tract [bp]** | **Longest tract [bp]** | **Co-conversion [bp]** | **Complex NCO [bp]** |
| --- | --- | --- | --- | --- | --- | --- |
| **9A/19A Ht** | 1027 | 908 ± 323 | 320 | 1806 | 336/1177 | 1177 |
|  | 1034 | 619 ± 294 | 129 | 1177 | 1048/1064 | 858/1177 |
|  | 1081 | 1281 ± 608 | 129 | 1806 | 1177 |  |
|  | 1391 | 1328 ± 1115 | 317 | 2983 |  | 2983 |
| **19A/19A Ho** | 1100 | 887 ± 335 | 670 | 1806 | 1177 | 1384 |
|  | 1227 | 1307 ± 431 | 670 | 1919 | 1065 | 1384 |
|  | 1251 | 829 ± 370 | 320 | 1919 | 1177 | 1919 |
|  | 1288 | 1104 ± 440 | 670 | 1806 |  |  |
| **9A/19A Ht** |  | 1004 ± 1113 | 129 | 2983 |  |  |
| **19A/19A Ho** |  | 1069 ± 585 | 320 | 2664 |  |  |
| **Mean** |  | 1037 ± 1264 | 225 | 2824 |  |  |
