# Supplementary material for: The impact of poly-A microsatellite heterologies in meiotic recombination
Source: Life Sci Alliance. 2019 Apr 25;2(2):e201900364. doi: 10.26508/lsa.201900364 (PMC6485458; doi:10.26508/lsa.201900364)
Supplement: Supplementary file 9 [file LSA-2019-00364_TableS8.docx]

**Supplement Table S8. Frequency of complex events per amplifiable sperm**

When calculating the frequency of complex events individually per donor group (Ht or Ho) and per CO and NCO, we observed significantly higher rates of complex NCO in Ht donors. We calculated the complex CO frequency as complex CO/amplifiable sperm/4. The NCO was calculated as complex NCO/amplifiable meiosis/4 (see also Supplement Figure S13). One meiotic division results in 4 sperm molecules.

|  |  | **Complex**  **events** | **Amplifiable sperm** | **Frequency** | **CI lower** | **CI upper** | **Odds Ratio** | **p** |
| --- | --- | --- | --- | --- | --- | --- | --- | --- |
| CO | 9A/19A Ht | 51 | 2,932,370 | 6.96E-05 | 5.18E-05 | 9.15E-05 | 0.80 | 0.4612 |
|  | 19A/19A Ho | 22 | 1,016,048 | 8.66E-05 | 5.43E-05 | 1.31E-04 |  |  |
| NCO | 9A/19A Ht | 19 | 124984 | 6.08E-04 | 3.66E-04 | 9.50E-04 | 2.98 | **<0.005** |
|  | 19A/19A Ho | 12 | 235490 | 2.04E-04 | 1.05E-04 | 3.56E-04 |  |  |
