# Supplementary material for: The impact of poly-A microsatellite heterologies in meiotic recombination
Source: Life Sci Alliance. 2019 Apr 25;2(2):e201900364. doi: 10.26508/lsa.201900364 (PMC6485458; doi:10.26508/lsa.201900364)
Supplement: Supplementary file 10 [file LSA-2019-00364_TableS9.docx]

**Supplement Table S9. Complex Crossovers**

Complex COs (CCO) are detected in ~70% of the cases upstream of the 9A/19A STR; whereas, CCO rates (CCO/CO) [%] between individual donor groups are nearly equal.

| **Donor** | **Type** | **Reciprocal** | **#CO** | **#CCO** | **CCO/CO [%]** | **95% Poisson CI [%]** | |  | **HTs** | **# of CCO per HT** |
| --- | --- | --- | --- | --- | --- | --- | --- | --- | --- | --- |
|  |  |  |  |  |  | **upper** | **lower** |  |  |  |
| 1027 | 9A/19A | RI | 475 | 17 | **3.58** | 5.99 | 2.25 |  | G-C-7a-a-a-19a-c-c-T-A | 2 |
|  |  |  |  |  |  |  |  |  | G-C-6a-a-c-19a-c-c-T-A | 6 |
|  |  |  |  |  |  |  |  |  | G-C-7a-g-c-19a-c-c-T-A | 2 |
|  |  |  |  |  |  |  |  |  | G-C-6a-g-c-19a-c-c-T-A | 4 |
|  |  |  |  |  |  |  |  |  | G-C-7a-a-c-19a-c-c-T-A | 2 |
|  |  |  |  |  |  |  |  |  | G-C-6a-g-a-19a-c-c-T-A | 1 |
|  |  | RII | 426 | 5 | **1.18** | 2.74 | 0.38 |  | C-T-7a-a-c-19a-t-t-C-C | 5 |
| 1034 | 9A/19A | RI | 749 | 9 | **1.20** | 2.28 | 0.55 |  | G-C-7a-a-c-9a-t-c-c-T-A | 5 |
|  |  |  |  |  |  |  |  |  | G-C-6a-a-c-19a-t-c-c-T-A | 2 |
|  |  |  |  |  |  |  |  |  | G-C-6a-g-c-19a-t-c-c-T-A | 1 |
|  |  |  |  |  |  |  |  |  | G-C-6a-a-c-19a-c-c-c-T-A | 1 |
|  |  | RII | 716 | 6 | **0.84** | 1.82 | 0.31 |  | C-T-6a-g-c-9a-c-t-t-C-C | 2 |
|  |  |  |  |  |  |  |  |  | C-T-6a-g-a-19a-c-t-t-C-C | 1 |
|  |  |  |  |  |  |  |  |  | C-T-6a-g-c-9a-t-t-t-C-C | 1 |
|  |  |  |  |  |  |  |  |  | C-T-7a-a-c-19a-c-t-t-C-C | 1 |
|  |  |  |  |  |  |  |  |  | C-T-6a-a-c-19a-c-t-t-C-C | 1 |
| 1081 | 9A/19A | RI | 302 | 4 | **1.31** | 3.35 | 0.36 |  | G-C-7a-a-c-9a-c-c-T-C | 4 |
|  |  | RII | 280 | 2 | **0.71** | 2.56 | 0.09 |  | C-T-6a-g-a-19a-t-t-C-A | 2 |
| 1391 | 9A/19A | RI | 81 | 3 | **3.70** | 10.82 | 0.76 |  | G-C-a-t-g-9a-c-t-t-C-C | 1 |
|  |  |  |  |  |  |  |  |  | G-C-g-t-a-19a-c-t-t-C-C | 2 |
|  |  | RII | 70 | 5 | **7.14** | 16.67 | 2.32 |  | C-T-g-c-a-19a-t-c-c-T-A | 3 |
|  |  |  |  |  |  |  |  |  | C-T-a-c-a-19a-t-c-c-T-A | 1 |
|  |  |  |  |  |  |  |  |  | C-T-a-t-g-19a-t-c-c-T-A | 1 |
| 1100 | 19A/19A | RI | 328 | 5 | **1.52** | 3.56 | 0.50 |  | G-C-7a-a-t-c-c-T-A | 3 |
|  |  |  |  |  |  |  |  |  | G-C-6a-g-c-c-c-T-A | 1 |
|  |  |  |  |  |  |  |  |  | G-C-6a-a-t-t-c-T-A | 1 |
|  |  | RII | 328 | 5 | **1.53** | 3.56 | 0.50 |  | C-T-6a-g-c-t-t-C-C | 3 |
|  |  |  |  |  |  |  |  |  | C-T-6a-g-t-t-t-C-C | 1 |
|  |  |  |  |  |  |  |  |  | C-T-6a-a-c-c-t-C-C | 1 |
| 1227 | 19A/19A | RI | 197 | 5 | **2.53** | 5.92 | 0.82 |  | G-C-6a-c-c-c-c-T-A | 2 |
|  |  |  |  |  |  |  |  |  | G-C-7a-a-t-c-c-T-A | 3 |
|  |  | RII | 192 | 0 | **0.000** | 1.92 | 0.00 |  |  |  |
| 1251 | 19A/19A | RI | 150 | 0 | **0.000** | 2.46 | 0.00 |  |  |  |
|  |  | RII | 149 | 7 | **4.71** | 9.68 | 1.89 |  | C-T-7a-g-a-c-t-c-T-A | 2 |
|  |  |  |  |  |  |  |  |  | C-T-7a-g-c-c-t-c-T-A | 4 |
|  |  |  |  |  |  |  |  |  | C-T-7a-g-a-t-c-c-T-A | 1 |
| 1288 | 19A/19A | RI | 224 | 0 | **0.000** | 1.65 | 0.00 |  |  |  |
|  |  | RII | 253 | 0 | **0.000** | 1.46 | 0.00 |  |  |  |
| **Total CCO** | 9A/19A |  | 3100 | 51 | **1.64** | 2.16 | 1.22 |  |  |  |
|  | 19A/19A |  | 1820 | 22 | **1.21** | 1.83 | 0.76 |  |  |  |
