# Supplementary material for: The impact of poly-A microsatellite heterologies in meiotic recombination
Source: Life Sci Alliance. 2019 Apr 25;2(2):e201900364. doi: 10.26508/lsa.201900364 (PMC6485458; doi:10.26508/lsa.201900364)
Supplement: Supplementary file 11 [file LSA-2019-00364_TableS10.docx]

**Supplement Table S10. Poly-A mutation frequencies**

*De novo* insertion/deletion mutation rates for poly-A repeats estimated from whole genome sequencing data of family pedigrees extracted from ([Fungtammasan et al., 2015](#_ENREF_31)) are shown ranging from 6As to 17As. Up to a length of 12 As there is an insertion bias, whereas for longer repeats the deletion rate is higher than the insertion rate.

|  | **Total #** | **# of Del** | **Deletion rate** | **# of Ins** | **Insertion rate** | **Total mutation frequency** | **In/del** |
| --- | --- | --- | --- | --- | --- | --- | --- |
| **A(6)** | 7259345 | 0 | 0.00E+00 | 63 | 8.68E-06 | 8.68E-06 | 63.00 |
| **A(7)** | 2911488 | 56 | 1.92E-05 | 61 | 2.10E-05 | 4.02E-05 | 1.09 |
| **A(8)** | 1018115 | 48 | 4.71E-05 | 71 | 6.97E-05 | 1.17E-04 | 1.48 |
| **A(9)** | 549021 | 77 | 1.40E-04 | 101 | 1.84E-04 | 3.24E-04 | 1.31 |
| **A(10)** | 312517 | 90 | 2.88E-04 | 115 | 3.68E-04 | 6.56E-04 | 1.28 |
| **A(11)** | 136172 | 70 | 5.14E-04 | 120 | 8.81E-04 | 1.40E-03 | 1.71 |
| **A(12)** | 49609 | 64 | 1.29E-03 | 77 | 1.55E-03 | 2.84E-03 | 1.20 |
| **A(13)** | 10169 | 24 | 2.36E-03 | 16 | 1.57E-03 | 3.93E-03 | 0.67 |
| **A(14)** | 963 | 5 | 5.19E-03 | 0 | 0.00E+00 | 5.19E-03 | 0.00 |
| **A(15)** | 51 | 1 | 1.96E-02 | 0 | 0.00E+00 | 1.96E-02 | 0.00 |
| **A(16)** | 4 | 0 | 0.00E+00 | 0 | 0.00E+00 | 0.00E+00 |  |
| **A(17)** | 1 | 0 | 0.00E+00 | 0 | 0.00E+00 | 0.00E+00 |  |
