# Supplementary material for: The impact of poly-A microsatellite heterologies in meiotic recombination
Source: Life Sci Alliance. 2019 Apr 25;2(2):e201900364. doi: 10.26508/lsa.201900364 (PMC6485458; doi:10.26508/lsa.201900364)
Supplement: Supplementary file 12 [file LSA-2019-00364_TableS11.docx]

**Supplement Table S11. Correction factors**

Here, the correction factors for each individual donor and CO, as well as NCO experiments, are shown. Multiplying the initial number of meiosis by the corresponding correction factor represents the amplifiable sperm.

| **State** | **Donor ID** | **CO experiments** | | | **NCO experiments** | | |
| --- | --- | --- | --- | --- | --- | --- | --- |
|  |  | **Meiosis** | **Correction factor** | **Amplifiable sperm** | **Meiosis** | **Correction factor** | **Amplifiable sperm** |
| 9A/19A | 1027 | 3,964,971 | 0.14 | 555,096 | 275,000 | 0.11 | 30,250 |
|  | 1034 | 6,680,000 | 0.22 | 1,469,600 | 274,140 | 0.10 | 27,414 |
|  | 1081 | 1,851,600 | 0.49 | 776,355 | 275,000 | 0.12 | 33,000 |
|  | 1391 | 938,000 | 0.14 | 131,320 | 312,000 | 0.11 | 34,320 |
| 19A/19A | 1100 | 1,920,000 | 0.19 | 364,800 | 275,000 | 0.35 | 96,250 |
|  | 1227 | 1,528,000 | 0.14 | 213,920 | 275,000 | 0.20 | 55,000 |
|  | 1251 | 1,826,400 | 0.10 | 182,640 | 312,000 | 0.17 | 53,040 |
|  | 1288 | 909,600 | 0.28 | 254,688 | 312,000 | 0.10 | 31,200 |
